# Impact of COVID‑19 pandemic restrictions and subsequent relaxation on the prevalence of respiratory virus hospitalizations in children

**DOI:** 10.1186/s12887-024-04566-9

**Published:** 2024-02-02

**Authors:** Ahmed Abushahin, Haneen Toma, Amal Alnaimi, Mutasim Abu-Hasan, Abdullah Alneirab, Hadeel Alzoubi, Antonisamy Belavendra, Ibrahim Janahi

**Affiliations:** 1 Department of Pediatric Medicine, Division of Pulmonology, Sidra Medicine, Doha, Qatar; 2 Weill Cornel Medicine-Qatar (WCM-Q), Doha, Qatar

**Keywords:** COVID-19, Respiratory infection, Bronchiolitis, RSV, Rhinovirus, Pandemic

## Abstract

**Background:**

The COVID-19 pandemic and the consequently adopted worldwide control measures have resulted in global changes in the epidemiology and severity of other respiratory viruses. We compared the number and severity of viral acute lower respiratory tract infection (ALRTI) hospitalizations and determined changes in causative respiratory pathogens before, during, and after the pandemic among young children in Qatar.

**Methods:**

In this single-center retrospective study, we reviewed data of children ≤ 36 months old who were admitted to Sidra Medicine in Qatar with a viral ALRTI during winter seasons (September–April) between 2019 and 2023. The study period was divided into three distinct seasons based on the pandemic-imposed restrictions as follows: (1) the period between September 2019 and April 2020 was considered the pre-COVID-19 pandemic season; (2) the periods between September 2020 and April 2021, and the period between January and April 2022 were considered the COVID-19 pandemic seasons; and (3) the periods between September 2022 and April 2023 was considered the post-COVID-19 pandemic season.

**Results:**

During the COVID-19 season, 77 patients were admitted, compared with 153 patients during the pre-COVID-19 season and 230 patients during the post-COVID-19 season. RSV was the dominant virus during the pre-COVID-19 season, with a detection rate of 50.9%. RSV infection rate dropped significantly during the COVID-19 season to 10.4% and then increased again during the post-COVID-19 season to 29.1% (*P* < 0.001). Rhinovirus was the dominant virus during the COVID-19 (39.1%) and post-COVID-19 seasons (61%) compared to the pre-COVID-19 season (31.4%) (*P* < 0.001). The average length of hospital stay was significantly longer in the post-COVID-19 season than in the pre-COVID-19 and COVID-19 seasons (*P* < 0.001). No significant differences in the pediatric intensive care unit (PICU) admission rate (*P* = 0.22), PICU length of stay (*p* = 0.479), or respiratory support requirements were detected between the three seasons.

**Conclusion:**

Our study showed reduced viral ALRTI hospitalizations in Qatar during the COVID-19 pandemic with reduced RSV detection. An increase in viral ALRTI hospitalizations accompanied by a resurgence of RSV circulation following the relaxation of COVID-19 restrictions was observed without changes in disease severity.

## Background

Respiratory syncytial virus (RSV) is the most frequent cause of viral acute lower respiratory tract infections (ALRTIs) manifesting as bronchiolitis and pneumonia in children, particularly those < 2 years old. The disease is associated with high morbidity and mortality [1, 2]. According to Ting et al., RSV is estimated to cause 33 million ALRTI cases annually in children < 5 years with 3.2 million hospitalizations and at least 60,000 in-hospital deaths [3]. Approximately 45% of these RSV hospitalizations and in-hospital deaths occur in infants < 6 months old [2, 3].

RSV infection usually shows a distinct seasonal pattern. In temperate areas, the RSV season begins in November, ends in March, and peaks in January and February [4, 5]. The RSV runs year-round in tropical countries, peaking during the rainy season [4, 5]. In Qatar, RSV is the most common viral infection in children, accounting for 45.7% of hospitalizations with acute bronchiolitis, while respiratory viruses other than RSV were found in 35.7% of cases [6]. Among those respiratory viruses, rhinovirus was detected in one-third of the cases, followed by adenovirus (14%), parainfluenza virus type 4 (14%), bocavirus (10%), coronavirus (7%), H1N1 (3.4%), parainfluenza virus type 1 (3.4%), parainfluenza virus type 2 (3.4%), and parainfluenza virus type 3 (3.4%). Furthermore, RSV exhibited a strong seasonal pattern with peak activity in October, November, December, and January. Despite seasonal peaks, RSV bronchiolitis can also infect young children throughout the year. In another study, RSV was detected in 51.2% of hospitalized children with acute bronchiolitis, followed by rhinovirus (25.5%), human metapneumovirus (6.2%) and parainfluenza virus type 3 (5.1%) [7].

On the other hand, Coronavirus disease (COVID-19) is a newly discovered worldwide disease caused by the severe acute respiratory syndrome coronavirus 2 (SARS-CoV-2). It first appeared in Wuhan in December 2019, with a subsequent rapid outbreak with substantially increased hospitalization and deaths globally [8]. The World Health Organization declared COVID-19 a pandemic on March 11, 2020 [9]. Subsequently, nonpharmacological interventions (NPIs), including lockdowns, mask-wearing, social distancing, closure of schools and daycare centers, and travel restrictions, have been introduced in many countries. Most countries adopted such measures between March and April of 2020 to reduce social contact and minimize the spread of SARS-CoV-2 [9].

However, NPIs during the global COVID-19 pandemic have affected the circulation of SARS-CoV-2 as well as most seasonal respiratory viruses, leading to a new epidemiology of respiratory infections worldwide [10–14]. The most evident effect was the substantial reduction in RSV cases since March 2020 in the Southern and Northern Hemispheres [15, 16]. During the COVID-19 pandemic, a decline of approximately 98% in RSV and 99.4% in influenza ALRTIs were reported in Australia throughout the autumn/winter season (March–September) of 2020, compared with the previous seasons between 2012 and 2019 [17]. Similarly, the reported hospitalizations for RSV and influenza-related ALRTIs have decreased in many countries [18, 19].

Along with the changes in RSV incidence, the seasonality of RSV epidemics has changed in many countries during the pandemic. Australia and France reported a delayed RSV peak compared with the usual peak in the winter seasons [20, 21]. In addition, the United States Center for Disease Control and Prevention has reported an out-of-season increase in RSV circulation between May and June 2021 [22].

The COVID-19 pandemic in Qatar was part of the global pandemic. The first case was confirmed on February 27, 2020 [23]. Starting on March 10, 2020, Qatar has taken preventive measures to control the COVID-19 pandemic and promote social distancing, which has contributed to limiting its spread. Together with school and daycare center closures, all non-essential public places were closed [24]. As a result, the community transmission of SARS-CoV-2 has decreased significantly, allowing for a gradual loosening of lockdown measures. Therefore, several restrictions were removed, and schools and daycare centers were reopened in October 2021 [25]. However, physical distancing, wearing masks in public areas, and hand washing continued to be recommended.

Concerns were raised regarding RSV’s increased incidence and severity when restrictive measures were relaxed, as a resurgence of RSV cases was reported from different regions [26–28].

The impact of COVID-19 restrictions and their subsequent relaxation on the incidence and severity of viral ALRTIs among children in Qatar remains poorly documented. This study aimed to compare the number and severity of viral ALRTI hospitalizations and determine the changes in causative respiratory pathogens, particularly RSV, before, during, and after the pandemic among young children.

## Methods

In this single-center retrospective observational study, we reviewed the data of patients admitted to Sidra Medicine, the main children’s hospital in Doha, Qatar, during all winter seasons (September to April) between 2019 and 2023. This study period was divided into three distinct seasons based on the pandemic imposed restrictions as follows: (1) the period between September 2019 and April 2020 was considered the pre-COVID-19 pandemic season, (2) the periods between September 2020 and April 2021 and between January and April 2022 were considered the COVID-19 pandemic season, during which the COVID-19-related restrictions were implemented, and (3) the period between September 2022 and April 2023 was considered the post-COVID-19 pandemic season, during which the COVID-19-related restrictions were lifted.

All children ≤ 36 months old who were admitted to Sidra Medicine between September and April each year between 2019 and 2023 and diagnosed with viral ALRTI were included in the study. Diagnoses of viral ALRTI were identified from the electronic hospital information system using the following codes: viral acute lower respiratory tract infection, bronchiolitis, and viral bronchiolitis.

Demographic data, including age, sex, and gestational age, as well as clinical data, including a history of bronchopulmonary dysplasia (BPD), congenital heart disease (CHD), and other comorbidities (Down syndrome, cystic fibrosis, primary ciliary dyskinesia, and cerebral palsy), were collected. Hospitalization-related data were also obtained from the electronic medical records, including length of hospitalization, admission to the pediatric intensive care unit (PICU), oxygen supply, and ventilation support.

According to commercially available kits, nasopharyngeal swabs were routinely used to detect respiratory viruses using polymerase chain reaction. The kit included the following common respiratory viruses: respiratory syncytial virus (RSV), adenovirus, parainfluenza virus 1–3, influenza virus A, influenza virus B, human metapneumovirus (HMPV), and rhinovirus.

We compared the number of viral ALRTI hospitalizations during the pre-COVID-19 season, COVID-19 season, and post-COVID-19 season. We also described the pattern of respiratory pathogens associated with viral ALRTI hospitalizations during the three seasons, particularly the proportion of RSV-related viral ALRTI in children ≤ 36 months old. Furthermore, we compared the severity of viral ALRTI between the three seasons using the following as measures of severity: length of hospital stay (LOHS), PICU admission, and respiratory support, including Oxygen via nasal cannula, high flow nasal cannula (HFNC) Oxygen, non-invasive and invasive mechanical ventilation.

### Statistical analysis

For descriptive analyses, mean and standard deviation or median (range) were used to summarize quantitative variables depending on the distribution. Frequencies and percentages were used for categorical variables. For inferential analyses, outcome variables such as length of hospitalization, PICU admission, a high-flow nasal cannula (HFNC), and other PICU variables, and viruses were compared by COVID-19 season (pre-COVID-19, COVID-19, and post-COVID-19) using box plots with the Kruskal‒Wallis test and Bonferroni test for pairwise comparisons and bar charts with the chi-square test. Multiple regression analysis was performed to determine risk factors for length of hospitalization (after log transformation), PICU admission, and HFNC, adjusting for age, RSV, rhinovirus, BPD, gastroesophageal reflux disease (GERD), other comorbidities, and COVID-19 season. Estimated odds ratios (ORs) with 95% confidence intervals (CIs) for all risk factors were reported. Statistical analyses were performed using the STATA software 17/SE (StataCorp. 2021. *Stata Statistical Software: Release 17*. College Station, TX, StataCorp LLC).

## Results

A total of 460 patients (264 males and 196 females) diagnosed with viral ALRTI were admitted to Sidra Medicine during the study period. The median (interquartile range [IQR]) age was 9.0 (4.3, 16.3) months. The median (IQR) gestational age was 38 (36, 39) weeks. The median birth weight (IQR) was 2900 (2300, 3200) grams. The patient demographic and clinical characteristics are summarized in Table 1.

**Table 1:** Demographic and clinical characteristics of the patients

Variables	Overall (*n* = 460)	Pre-COVID-19 (*n* = 153)	COVID-19 (*n* = 77)	Post-COVID-19 (*n* = 230)	***P*** value†
Age (months), median (IQR)	9.0 (4.3, 16.2)	7.0 (3.1, 14.7)	8.6 (4.7, 15.1)	10.4 (4.7, 17.0)	0.022
Male, n (%)	264 (57.4)	91 (59.5)	46 (59.7)	127 (55.2)	0.641
Birth weight (grams), median (IQR)	2900 (2300, 3200)	3000 (2270, 3300)	2950 (2000, 3200)	2890 (2300, 3100)	0.726
Gestational age (weeks), median (IQR)	38 (36, 39)	38 (35, 39)	38 (36, 39)	38 (36, 39)	0.873
Multiple births, n (%)	33 (7.2)	18 (11.8)	3 (3.9)	12 (5.2)	0.025
**Comorbidities**					
BPD, n (%)	37 (8.0)	11 (7.2)	9 (11.7)	17 (7.4)	
None	423 (92.0)	142 (92.8)	68 (88.3)	213 (92.6)	
Mild	12 (2.6)	2 (2.6)	2 (2.6)	8 (3.5)	0.237
Moderate	11 (2.4)	6 (3.9)	3 (3.9)	2 (0.9)	
Severe	14 (3.0)	3 (2.0)	4 (5.2)	7 (3.0)	
Congenital heart disease, n (%)	145 (31.5)	34 (22.2)	30 (38.9)	81 (35.2)	
None	315 (68.5)	119 (77.8)	47 (61.0)	149 (64.8)	
Acyanotic heart disease	105 (22.8)	28 (18.3)	27 (35.1)	50 (21.7)	< 0.001
Cyanotic heart disease	40 (8.7)	6 (3.9)	3 (3.9)	31 (13.5)	
GERD, n (%)	89 (19.4)	17 (11.1)	27 (35.1)	45 (9.6)	< 0.001
Other comorbidities, n (%)	189 (41.1)	39 (25.5)	34 (44.2)	116 (50.4)	< 0.001

Median (IQR) for non-normally distributed variables; ^†^*P* value is obtained using the Kruskal-Wallis test for non-normally distributed continuous variables and chi-square test for categorical variables

BPD: bronchopulmonary dysplasia; GERD: gastroesophageal reflux disease; other comorbidities: down syndrome, cystic fibrosis, primary ciliary dyskinesia, cerebral palsy

During the whole study period, at least one virus was detected in 89.1% of the patients and more than one virus in 22.6%. Multiple viruses were detected in 34% of patients during the post-COVID-19 season, compared with 18% during the pre-COVID-19 season and 28% during the COVID-19 season (*P* < 0.001). Significant changes in viral distribution over the three periods were also observed. RSV was the dominant virus in the pre-COVID-19 season, with a detection rate of 50.9% (78/153), but dropped significantly in the COVID-19 and post-COVID-19 seasons to 10.4% (8/77) and 29.1% (67/230), respectively (*P* < 0.001). Rhinovirus was the dominant virus in the COVID-19 and post-COVID-19 seasons compared with that in the pre-COVID-19 season and was detected in 39.1% (90/230), 61% (47/77), and 31.4% (48/153) (*P* < 0.001), respectively. Human metapneumovirus was higher in the COVID-19 and post-COVID-19 seasons than in the pre-COVID-19 season (*P* < 0.02). Influenza A virus was evident in the post-COVID-19 season in 14 patients, compared with only one patient in the pre-COVID-19 season and none in the COVID-19 season (*P* < 0.003). The viral distribution in the three seasons is shown in Table 2.

**Table 2:** Epidemiological changes in circulating respiratory viruses during the study period

Variables	Overall (*n* = 460)	Pre-COVID-19 (*n* = 153)	COVID-19 (*n* = 77)	Post-COVID-19 (*n* = 230)	***P*** value
RSV, n (%)	153 (33.3)	78 (51.0)	8 (10.4)	67 (29.1)	< 0.001
Rhino, n (%)	185 (40.2)	48 (31.4)	47 (61.0)	90 (39.1)	< 0.001
Adeno, n (%)	31 (6.7)	7 (4.6)	8 (10.4)	16 (7.0)	0.248
HMPV, n (%)	40 (8.7)	8 (5.2)	12 (15.8)	20 (8.7)	0.028
Corona, n (%)	13 (2.8)	6 (3.9)	2 (2.6)	5 (2.2)	0.595
Parainfluenza, n (%)	44 (9.6)	11 (7.2)	7 (9.2)	26 (11.4)	0.397
Influenza A, n (%)	15 (3.3)	1 (0.7)	0 (0.0)	14 (6.1)	0.003
Boca, n (%)	2 (0.4)	1 (0.7)	0 (0.0)	1 (0.4)	0.777
Norovirus, n (%)	1 (0.2)	1 (0.7)	0 (0.0)	0 (0.0)	0.366
Influenza B, n (%)	3 (0.7)	1 (0.7)	0 (0.0)	2 (0.9)	0.714
Parecho, n (%)	1 (0.2)	1 (0.7)	0 (0.0)	0 (0.0)	0.366

During the COVID-19 season, 77 patients were admitted, compared with 153 patients during the pre-COVID-19 season and 230 patients during the post-COVID-19 season. The median age of hospitalized patients was 7 months (IQR 3.1, 14.7) during the pre-COVID-19 season, 8.6 months (IQR 4.7–15.1) during the COVID-19 season, whereas the median age was higher during the post-COVID season (10.5 months, IQR 4.7–17.0) (*P* < 0.022). During the COVID-19 and post-COVID-19 seasons, patients with existing congenital heart disease were 38.9% and 35%, respectively, compared with 22.2% in the pre-COVID-19 season (*P* < 0.001) (Table 1).

**Fig. 1:**
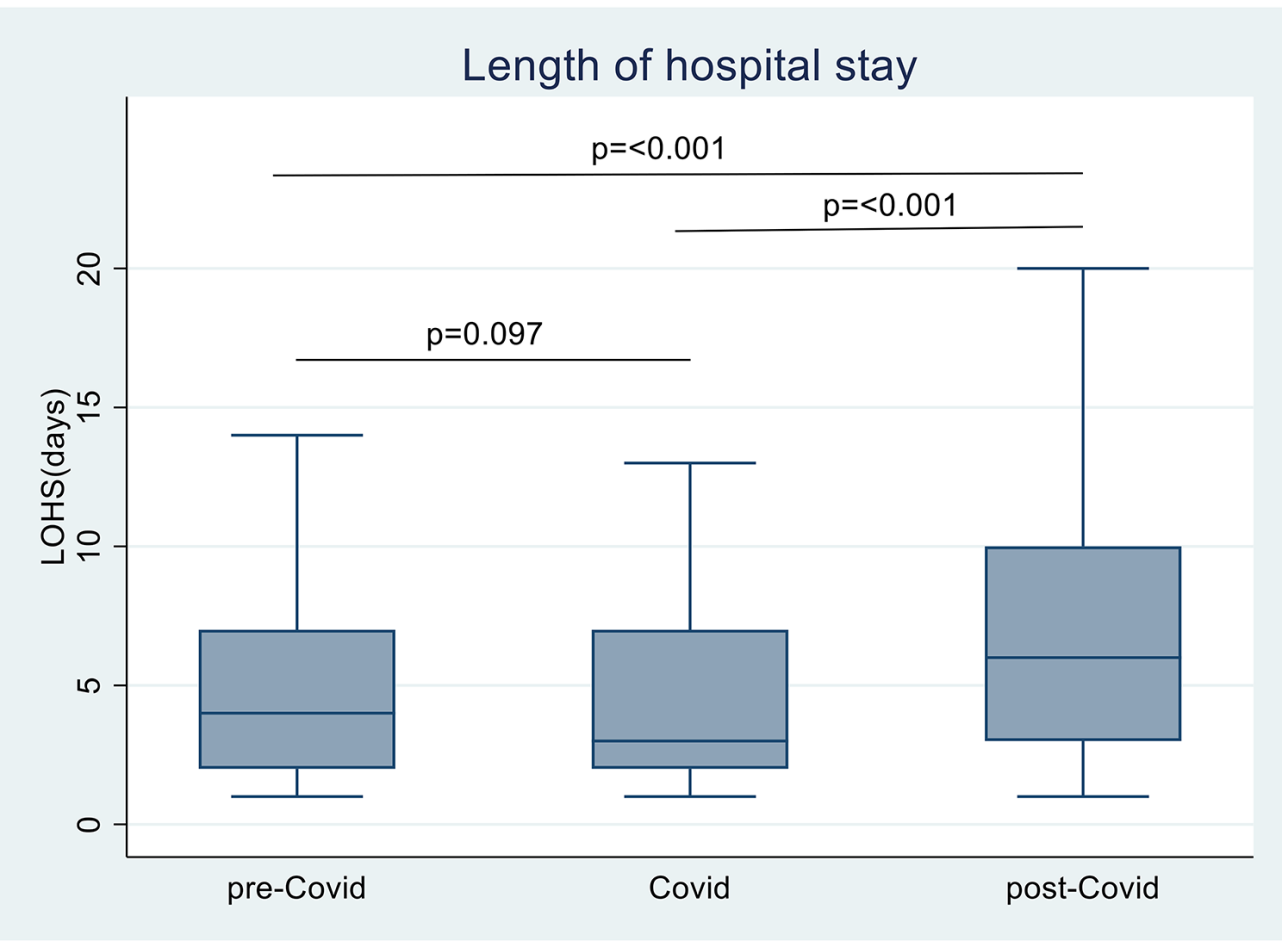
Average length of hospital stay during the three study periods

The average (range) length of hospital stay was significantly longer in the post-COVID-19 season, with a median (range) of 8 (1–44) days than in the pre-COVID-19 and COVID-19 seasons, with median (range) lengths of 7 (1–89) and 7 (1–59) days, respectively (*P* < 0.001) (Fig. 1).

Among all patients, 107 (23.3%) required PICU admission. The average (range) PICU length of stay was 6 (1–30) days. No significant differences were observed in the PICU admission rate or length of stay among the three seasons (*P* = 0.22, *P* = 0.479, respectively) (Table 3). No significant differences in respiratory support requirements were detected among patients in the three seasons (Table 3).

**Table 3:** Impact of the pandemic on viral ALRTI-disease severity during pre-COVID-19, COVID-19, and post-COVID-19 seasons

Variables	Total	Pre-COVID-19	COVID-19	Post-COVID-19	***P*** value
PICU admissions, n (%)	107 (23.2%)	43 (28.1)	16 (20.7)	48 (20.8)	0.222
Oxygen, n (%)	10 (2.2)	5 (3.3)	1 (1.3)	4 (1.7)	0.505
HFNC, n (%)	36 (7.8)	17 (11.1)	5 (6.5)	14 (6.1)	0.182
NIV, n (%)	39 (8.5)	16 (10.5)	7 (9.1)	16 (7.0)	0.473
MV, n (%)	24 (5.2)	6 (3.9)	2 (2.6)	16 (7.0)	0.224
PICU stay (days), median (range)	6 (1, 30)	4 (1, 20)	4 (1, 30)	5 (1, 22)	0.479
PPV (days), median (range)	2 (1, 36)	2 (1, 14)	2 (1,36)	2 (1, 18)	0.950

Median (range) for non-normally distributed continuous variables

HFNC: high-flow nasal cannula; NIV: non-invasive ventilation; MV: mechanical ventilation; PPV: positive pressure ventilation

Multiple regression analysis showed young age (OR 0.95, CI: 0.92, 0.99; *P* = 0.005) and history of comorbidities other than CHD and BPD as significant predictors of PICU admissions (OR 2.43, CI: 1.4, 4; *P* = 0.001) (Table 4). Hospital stay was significantly longer in patients with GERD (OR 1.52, CI: 1.2, 1.9; *P* < 0.001) and patients with comorbidities (OR 1.41, CI: 1.2, 1.7; *P* < 0.001) (Table 4). HFNC was more likely to be required in younger patients (OR 0.95, CI: 0.92, 0.98; *P* = 0.001) (Table 4).

The severity of RSV-ALRTI, as indicated by the length of hospital stay, PICU admission rate, and need for invasive, non-invasive, or HFNC, was not significantly different between the three seasons (Table 4).

**Table 4:** Multiple regression analysis of the risk factors for LOHS, PICU admissions, and need for HFNC

	Logged LOHS		PICU		HFNC	
Variables	e ^β^ (95% CI)	***P*** value	OR (95% CI)	***P*** value	OR (95% CI)	***P*** value
Age, months	0.99 (0.98, 1.0)	0.372	0.95 (0.92, 0.99)	0.005	0.95 (0.92, 0.98)	0.001
RSV (yes)	1.12 (0.9, 1.4)	0.283	1.16 (0.7, 2.0)	0.600	1.27 (0.8, 2.1)	0.364
Rhino (yes)	0.92 (0.8, 1.1)	0.359	1.30 (0.8, 2.2)	0.318	0.70 (0.4, 1.1)	0.160
BPD (yes)	0.96 (0.7, 1.3)	0.817	0.65 (0.2, 1.8)	0.407	1.32 (0.6, 2.9)	0.493
GERD (yes)	1.52 (1.2, 1.9)	< 0.001	1.28 (0.7, 2.3)	0.394	1.00 (0.6, 1.8)	0.983
Comorbidity (yes)	1.41 (1.2, 1.7)	< 0.001	2.43 (1.4, 4.1)	0.001	1.40 (0.8, 2.3)	0.189
COVID-19 season						
Pre-COVID-19 Post-COVID-19	1.29 (0.99, 1.7) 1.61 (1.3, 2.0)	0.061 < 0.001	1.91 (0.9, 3.9) 1.06 (0.5, 2.1)	0.077 0.870	0.85 (0.4, 1.7) 1.01 (0.5, 1.9)	0.648 0.984

Effect size estimates are presented as odds ratios and 95% confidence intervals as exponentiated β-coefficients (e^β^) for the logged LOHS. The models were adjusted for age, RSV, Rhino, BPD, GERD, other comorbidities, and the COVID season. LOHS: Length of hospital stay; PICU: Pediatric intensive care unit; HFNC: High flow nasal cannula

## Discussion

Our study revealed a decline in the total number of children hospitalized with viral ALRTIs during the COVID-19 season (77 patients) compared with that during the pre-COVID-19 season (153 patients), indicating a definite impact of the lockdown in Qatar on decreasing viral ALRTI hospitalizations, as reported in other countries [5, 29]. A study from Spain reported a substantial reduction in bronchiolitis admissions, reaching 62% in 2020 compared with that in 2016–2019 [30]. Another study from China reported a decline in viral ALRTI hospitalizations by 23.2% and 28.0% in 2020 compared with those in 2018 and 2019, respectively [19].

Pandemic-associated changes in the epidemiology of circulating respiratory viruses have been reported worldwide [31, 32]. The RSV detection rate in our cohort decreased from 51% during the pre-COVID-19 season to 10.4% during the COVID-19 season. In contrast, rhinovirus detection increased from 31.4% during the pre-COVID-19 season to 61% during the COVID-19 season. Similar to our findings, a study from Spain showed a 65.9% reduction in RSV detection in 2020 compared with that in 2016–2019 [30]. In addition, a reduction rate for RSV acute bronchiolitis of − 44.3 (95% CI − 73.8 to − 14.8) has been observed by Reyes Dominguez et al. [33]. Furthermore, our study showed a predominance of rhinovirus detection during the COVID-19 pandemic season, as reported by others [19, 20]. A recent report from China showed a significant increase in rhinovirus infection in 2020, more evident after school reopening [16]. This finding suggests that the pandemic has dramatically impacted the circulating respiratory viruses, with differential effects on RSV and rhinovirus.

The exact mechanism for the observed changes in circulating respiratory pathogens is not entirely understood. One possible and obvious explanation is the introduction of restrictions during the pandemic [13, 32]. Furthermore, the viral characteristics, communicability, and transmission mode vary depending on the pathogen variants and subvariants, which can also explain the epidemiological differences [32]. Non-enveloped viruses, such as rhinovirus and human adenovirus, are usually more virulent, have extended shedding, and are resistant to simple disinfection measures [34]. Therefore, they are minimally affected and maintain their usual circulatory levels despite implementing the NPIs during the pandemic. In contrast, enveloped viruses, such as RSV and influenza, are more sensitive and can be significantly reduced after NPI implementation [32, 34]. This could explain the near disappearance of RSV during COVID-19 and the return of RSV to pre-pandemic levels after the easing of NPIs.

Previous studies have reported increased viral ALRTI hospitalizations after relaxing COVID-19 restrictions [33, 35]. Similarly, we found an increase in viral ALRTI hospitalizations in the post-COVID-19 season (230 patients) compared with that during the COVID-19 season (77 patients). Our study showed a substantial increase in RSV cases, reaching 29.1% after relaxation compared with that reported during the COVID-19 lockdown (10.4%), but did not reach the detection level seen during the pre-COVID-19 season (51%). Similarly, Foley et al. reported a substantial increase in RSV cases in Western Australia after relaxing physical distancing compared to previous seasons [20]. In addition to the relaxation effects, the possible appearance of new viral strains that might occur during the COVID-19 pandemic could increase hospitalization. Furthermore, the absence of viral exposure during the pandemic decreases the protective immune response [32]. This immunity gap makes children particularly vulnerable to viral respiratory infections [5]. Our study showed a significant reduction in the detection of rhinovirus cases from 61% during the COVID-19 season to 39.1% after the relaxation, which coincided with the increase in RSV detection. However, it remained higher than that observed in the pre-COVID-19 season (31.4%). In contrast, Redlberger-Fritz et al. Observed a significant reduction of influenza virus, RSV, HMPN, and rhinovirus cases after the lockdown in 2020, compared to the previous five seasons in Austria. They reported a surge in rhinovirus cases after the relaxation of multiple lockdown measures, surpassing the same period in the early years [36].

Our study demonstrated that patients were younger in the pre-COVID-19 and COVID-19 seasons than in the post-COVID-19 seasons. In addition, the average length of hospital stay was significantly longer in the post-COVID-19 season than in the pre-COVID-19 and COVID-19 seasons. The extended hospitalization and older age group seen in our study post-relaxation of restrictions can be attributed to a lack of immunity and inadequate previous exposure. However, our study showed no significant differences in the PICU admission rate, level of respiratory support, or length of stay between the three study periods. A study in the United States showed a resurgence of RSV post-pandemic with more severe disease [37].

In contrast, Torres-Fernandez et al. showed a significant decrease in bronchiolitis admissions to the PICU during the pandemic compared with pre-pandemic seasons [30]. Furthermore, Fourgeaud et al. reported a less severe form of RSV-ALRTI affecting relatively older infants during the pandemic than in previous seasons [18]. These inconsistent findings could be partly explained by variations in the immune response of the vulnerable host, combined with changes in the characteristics of respiratory viruses that may have occurred during and post-COVID-19 pandemic.

In the multiple regression analysis, young age and comorbidities other than CHD and BPD were significantly correlated with PICU admission. In contrast, RSV infection did not correlate with severe viral respiratory illnesses. These results suggest that pandemic-related restrictions and subsequent relaxation did not influence RSV virulence in our cohort.

This is the only study in Qatar that provides a national perspective on the epidemiology of respiratory pathogens related to the COVID-19 pandemic. The study has limitations due to being a retrospective from a single-center study and being focused on hospitalized patients with severe viral ALRTIs. Therefore, the conclusions drawn from the study may not represent the viral ALRTI epidemiology in the larger population. In future respiratory viral pandemics, large multicenter epidemiological studies are warranted to delineate the epidemiology of respiratory virus circulation in Qatar. This helps implement appropriate preventive and potential management strategies for these viral respiratory illnesses.

## Conclusion

Our study showed reduced viral ALRTI hospitalizations during the COVID-19 pandemic, mainly due to reduced RSV circulation, with a subsequent resurgence of RSV after the pandemic. Our findings contribute to increasing awareness of the increased appearance of RSV during the post-COVID-19 winter season so that prevention and management strategies can be modulated accordingly.

## Data Availability

The data that support these findings are available on request from the corresponding author.

## Abbreviations

RSV: respiratory syncytial virus
ALRTI: acute lower respiratory tract infection
NPIs: nonpharmacological interventions
SARS-CoV-2: severe acute respiratory syndrome coronavirus 2
BPD: bronchopulmonary dysplasia
PICU: pediatric intensive care unit
GERD: gastroesophageal reflux disease
LOHS: Length of hospital stay
HFNC: high-flow nasal cannula
NIV: non-invasive ventilation
MV: mechanical ventilation
PPV: positive pressure ventilation
